# ﻿A new species and two new distributional records of Signiphoridae (Hymenoptera, Chalcidoidea) from the canopy of the Xishuangbanna Rainforest

**DOI:** 10.3897/zookeys.1264.157313

**Published:** 2025-12-11

**Authors:** Hui-Feng Zhao, Hui Geng, Hai-Feng Chen, Ye Chen

**Affiliations:** 1 Hebei Key Laboratory of Animal Diversity, College of Life Science, Langfang Normal University, Langfang, 065000, China Langfang Normal University Langfang China; 2 College of Life Sciences, Shangrao Normal University, Shangrao, 334001, China Shangrao Normal University Shangrao China

**Keywords:** *
Chartocerus
*, *
Signiphora
*, parasitic wasps, taxonomy

## Abstract

Three species of Signiphoridae are recorded from the Xishuangbanna Rainforest (China, Yunnan). *Chartocerus menglaensis***sp. nov.** is described as new to science. *Chartocerus niger* (Ashmead) and *Signiphora flavella* Girault are recorded from China for the first time. A key to all Chinese species of Signiphoridae is provided.

## ﻿Introduction

Signiphoridae is a small family of Chalcidoidea and includes 88 extant species in four genera (UCD 2025). Most species of this family belong to the genus *Chartocerus* Motschulsky or *Signiphora* Ashmead with 37 and 46 species, respectively, and have a worldwide distribution. *Thysanus* Walker has four species, with *T. ater* widely distributed and other three reported from the USA or Peru. *Clytina* Erdös has one species from Europe and Central Asia. The species of this family are parasitoids and hyperparasitoids associated with a variety of insect hosts, but mostly scale insects, mealybugs, and their predators (Woolley 1988).

The generic and family-level nomenclature of Signiphoridae were confused for a long time until Woolley’s (1986, 1988) great contribution to the group, which stabilised the current classification of the family. There are many other important taxonomic works on the group. For example, Girault (1913) provided the taxonomic history of the group, which was then treated as a subfamily (containing only the genus *Signiphora*) within Encyrtidae, and studied all species known at that time; Rosanov (1965) reviewed the genera of Signiphoridae and provided a key; Quezada et al. (1973) provided descriptions and illustrations of all life stages of *Signiphora borinquensis* Quezada, DeBach & Rosen, making it probably the most detailed treatment of the biology of any signiphorid species; De Santis (1973) studied the Signiphoridae from Argentina; Hayat (2009) reviewed the Indian Signiphoridae, Woolley and Dal Molin (2017) revised the *Signiphora flavopalliata* species group worldwide; Schmidt et al. (2019) reviewed the species from Indonesia; Dal Molin and Woolley (2020) redescribed and illustrated some type material of Australian *Chartocerus*, which were described in the early 20^th^ century.

The Chinese signiphorid fauna is poorly known and includes only seven species: *Chartocerus fujianensis* Tang, *C. musciformis* Motschulsky, *C. subaeneus* (Foerster), *C. walkeri* Hayat, *C. yunnanensis* Tan & Zhao, *Signiphora merceti* Malenotti, and *Thysanus ater* Walker (Rosanov 1965; Tang 1985; Tan and Zhao 1995; Xu and Huang 2004; Wang et al. 2019; Geng et al. 2023). Herein, we add a new species and two new distributional records of this group from the Xishuangbanna Rainforest in Yunnan Province.

## ﻿Material and methods

Samples were obtained using pyrethroid fog generated from a thermal fogger (Swingfog SN50, Germany, Model 2610E, Series 3) in the rainforest canopy at the Xishuangbanna Tropical Botanical Garden in Menglun Town, Yunnan Province. Each fogging session lasted about 30 minutes. After waiting for 1 hour, all the samples fell into 1 m^2^ collection trays, which were suspended 1.5 m above the ground. The individuals of Signiphoridae were sorted out using stereomicroscope (stemi 305, Zeiss) in the laboratory. The specimens were dissected and mounted on slides in Canada balsam, following the method described by Woolley and Dal Molin (2017). The photographic methods followed Chen and Chen (2021). Scale bars are 100 μm except where otherwise indicated. All specimens listed below are deposited in Langfang Normal University, Langfang, China.

Terminology follows the chapter 5 of “Chalcidoidea of the World” (Heraty and Woolley 2025) for most parts of the body, but Woolley (1988) for the setae on the marginal vein (the setae on the marginal vein are numbered as M1 to M6, cf. figs 19–21 in Woolley 1988). The following abbreviations are used in the text: Mt_1_, Mt_2_, etc., tergites 1, 2, etc. of metasoma.

### ﻿Abbreviations for depositories

**LFNU** Langfang Normal University, Langfang, China;

**USNM** Smithsonian National Museum of Natural History, Washington, DC, USA

## ﻿Taxonomy

### ﻿Key to the species of Signiphoridae from China

**Table:** 

1	Protibial spur with comb of fine setae (cf. fig. 28 in Woolley 1988), propodeum with lamelliform process posteriorly (cf. fig. 10 in Woolley 1988), antenna of female with 3 anelli (genus *Signiphora*)	**2**
–	Protibial spur without comb of fine setae (cf. fig. 27 in Woolley 1988), propodeum without lamelliform process posteriorly, antenna of female with 4 anelli	**3**
2	Body uniformly brown, fore wing infuscate from base to apex, except a hyaline area on posterobasal surface (cf. fig. 268B in Xu and Huang 2004)	***Signiphora merceti* Malenotti**
–	Body with most of mesosoma pale yellow, fore wing infuscate from base to posterior to stigmal vein except a hyaline area on posterobasal surface (fig. 27)	***S. flavella* Girault**
3	Fore wing with 4 setae on marginal vein, without M6 and M2b; hind wing with parallel margins; mesofemur with 1 long spine	***Thysanus ater* Walker**
–	Fore wing with 6 or 7 setae on marginal vein, M2b and M6 present; hind wing with posterior margin rounded; mesofemur with 3 or 4 long spines. (genus *Chartocerus*)	**4**
4	Longest marginal setae of fore wing more than 0.5× as long as width of fore wing	**5**
–	Longest marginal setae of fore wing less than 0.5× as long as width of fore wing	**6**
5	Mesotibia yellow, mesotibial spur with 6 teeth	***Chartocerus fujianensis* Tang**
–	Mesotibia dark, mesotibial spur with 9 teeth	***Chartocerus yunnanensis* Tan & Zhao**
6	Fore wing hyaline, the second to the fourth anellus nearly equal in length (cf. fig. 2 in Rosanov 1969 and fig. 27 in Hayat 2009)	**7**
–	Fore wing more or less infuscate, the fourth anellus obviously longer than the preceding two anelli	**8**
7	Pedicel 3.5× as long as wide	***Chartocerus walkeri* Hayat**
–	Pedicel 1.8× as long as wide (cf. fig. 2 in Rosanov 1969)	***C. musciformis* Motschulsky**
8	Fore wing largely infuscate, with a hyaline band medially (cf. fig. 19 in Rosanov 1965)	***C. subaeneus* (Foerster)**
–	Fore wing infuscate basally	**9**
9	Fore wing with transverse infuscation forming a band posterior to submarginal vein (Fig. 6), clava 5.2–6.2× as long as wide, fore wing 2.6–2.7× as long as wide, hind wing 3.3–3.6× as long as wide, posterior margin strongly rounded (Fig. 7), Mt_1_ with posterior margin almost straight medially (Fig. 5)	***C. menglaensis* sp. nov.**
–	Fore wing mostly infuscate on basal surface (Fig. 16), clava 4.7× as long as wide, fore wing 2.9× as long as wide, hind wing 4.0–4.3× as long as wide, posterior margin slightly rounded (Fig. 17), Mt_1_ with posterior margin concave medially (Fig. 15)	***C. niger* (Ashmead)**

#### ﻿ Chartocerus menglaensis

Taxon classificationAnimaliaHymenopteraSigniphoridae

Chen & Zhao sp. nov.

72860392-3007-5A05-802F-14266FF10AB1

https://zoobank.org/C87D9621-CF76-4B9A-9674-C2CDFA9FEF21

Figs 1–9

##### Type material.

***Holotype***: China • ♀; Yunnan Province; Xishuangbanna; Mengla County; Menglun Town; 21°53.92'N, 101°16.1'E; 560 m a.s.l.; 3 May. 2019; Z.-l. Bai, Z.-g. Chen, C. Wang, Yanfeng Tong, H. Yu leg.; LFNU Sc20210701 [on slide]. ***Paratypes***: • 1 ♀ Yunnan Province; Xishuangbanna; Mengla County; Menglun Town; 21°53.59'N, 101°17.29'E; 606 m a.s.l.; 4 May. 2019; Z.-l. Bai, Z.-g. Chen, C. Wang, Yanfeng Tong, H. Yu leg.; LFNU Sc20210702 [on slide]; • 1 ♀ Yunnan Province; Xishuangbanna; Mengla County; Menglun Town; 21°54.37'N, 101°16.71'E; 623 m a.s.l.; 6 May. 2019; Z.-l. Bai, Y.-j. Lin, C. Wang, Yanfeng Tong, H. Yu leg.; LFNU Sc20210703 [on slide].

**Figures 1–9.**
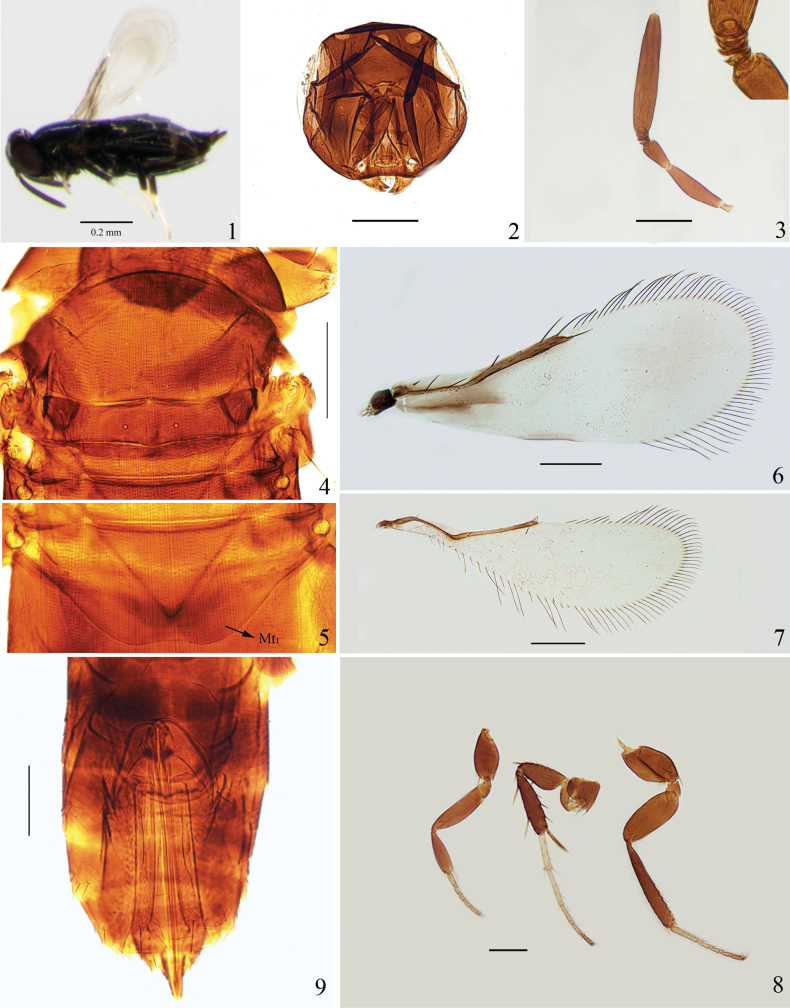
*Chartocerus menglaensis* sp. nov., ♀. **1.** Body, lateral view; **2.** Head, frontal view; **3.** Antenna (inset shows anelli); **4.** Mesosoma; **5.** Propodeum and Mt_1_; **6.** Fore wing; **7.** Hind wing; **8.** Legs; **9.** Metasoma. Scale bars: 100 μm (except Fig. 1).

##### Diagnosis.

Body black; fore wing (Fig. 6) mostly hyaline, narrowly infuscate posterior to submarginal vein. Clava 5.2–6.2× as long as wide; fore wing 2.6–2.7× as long as wide, with longest setae of marginal fringe 0.3× as long as width of disc; Hind wing (Fig. 7) 3.3–3.6× as long as wide, with posterior margin strongly rounded; Mt_1_ with posterior margin almost straight medially.

##### Description.

**Female.** Body length 0.8–0.9 mm.

***Colour*** (Fig. 1). Head and body bright black with bronze reflection; eyes dark red; antenna dark brown; legs mostly black, with tarsomeres yellowish brown except that 1–4 of mid leg and 1–2 of hind leg which are pale yellow; fore wing (Fig. 6) nearly hyaline, infuscate posterior to submarginal vein forming a narrowly transverse brown band, and infuscate along middle part of posterior margin; hind wing hyaline.

***Head*** (Fig. 2). In frontal view, 0.8× as high as wide, mostly smooth, with weakly lineolate sculpture on gena. Vertex two-thirds the width of head. Eye 0.6× as high as head. Mandible with two teeth. Antenna (Fig. 3) with four anelli; scape 3.8–4.9× as long as wide; pedicel 1.8–2.3× as long as wide; clava 5.2–6.2× as long as wide, 1.7–1.9× as long as scape.

***Mesosoma*** (Fig. 4). Pronotum, mesoscutum, and anterior of metanotum transversely imbricate; lateral sides of mesoscutellum and axillae reticulate. Mesoscutum 3.0× as long as mesoscutellum, with 13 setae posteriorly and a pair of long setae near posterolateral corner. Mesoscutellum with 10 setae along posterior margin, the distance of scutellar sensilla 0.26× the anterior width of mesoscutellum. Metanotum a little shorter than mesoscutellum. Mesopostphragma extending to posterior of Mt_4_.

***Wings*.** Fore wing (Fig. 6) 2.6–2.7× as long as wide, with longest setae of marginal fringe 0.3× as long as width of disc, seta M1 absent. Hind wing (Fig. 7) 3.3–3.6× as long as wide, bearing one seta posterior to the apex of marginal vein, posterior margin strongly rounded, with longest setae of marginal fringe 0.3× wing width.

***Legs*** (Fig. 8). Mid leg with basitarsus 0.5× as long as tibia, and the basitarsus nearly as long as mesotibial spur; mesotibial spur with 6–8 teeth.

***Metasoma*.** Mt_1_ with posterior margin almost straight medially (Fig. 5). Ovipositor originating from apex of Mt_3_, 2.3–2.6× as long as mesotibia, 1.7–1.8× as long as metatibia. Third valvula equal in length to mesobasitarsus.

**Male.** Unknown.

##### Host.

Unknown.

##### Etymology.

The specific name refers to the type locality.

##### Distribution.

Known only from Menglun town, Mengla county, Yunnan Province, China.

##### Comments.

This new species is similar to *Chartocerus niger* (Ashmead); the differences are provided in the key.

#### ﻿ Chartocerus niger

Taxon classificationAnimaliaHymenopteraSigniphoridae

(Ashmead, 1900)

7E6886D0-193B-5B07-84D0-48A960677BDE

Figs 10–19

Signiphora nigra Ashmead, 1900: 410. ♀, USNM, not examined.
Thysanus niger (Ashmead): Armitage 1923: 513.
Chartocerus niger (Ashmead): Rosanov 1965: 878; Woolley 1988: 492; Abd-Rabou and Evans 2016: 146.

##### Material examined.

• 2 ♀, Yunnan Province; Xishuangbanna; Mengla County; Menglun Town; 21°53.89'N, 101°16.72'E; 568 m a.s.l.; 12 May. 2019; Z.-l. Bai, Z.-g. Chen, C. Wang, H. Yu leg.; LFNU Sc20210704, Sc20210705 [on slides].

##### Diagnosis.

Body black; fore wing (Fig. 16) infuscate basally with a transverse hyaline area; clava 4.7× as long as wide; fore wing 2.9× as long as wide, with longest setae of marginal fringe 0.3× as long as width of disc, seta M1 absent; hind wing (Fig. 17) 4.0–4.3× as long as wide, bearing a seta posterior to the apex of marginal vein, posterior margin slightly rounded; Mt_1_ (Fig. 15) with posterior margin concave medially.

##### Description.

**Figures 10–19.**
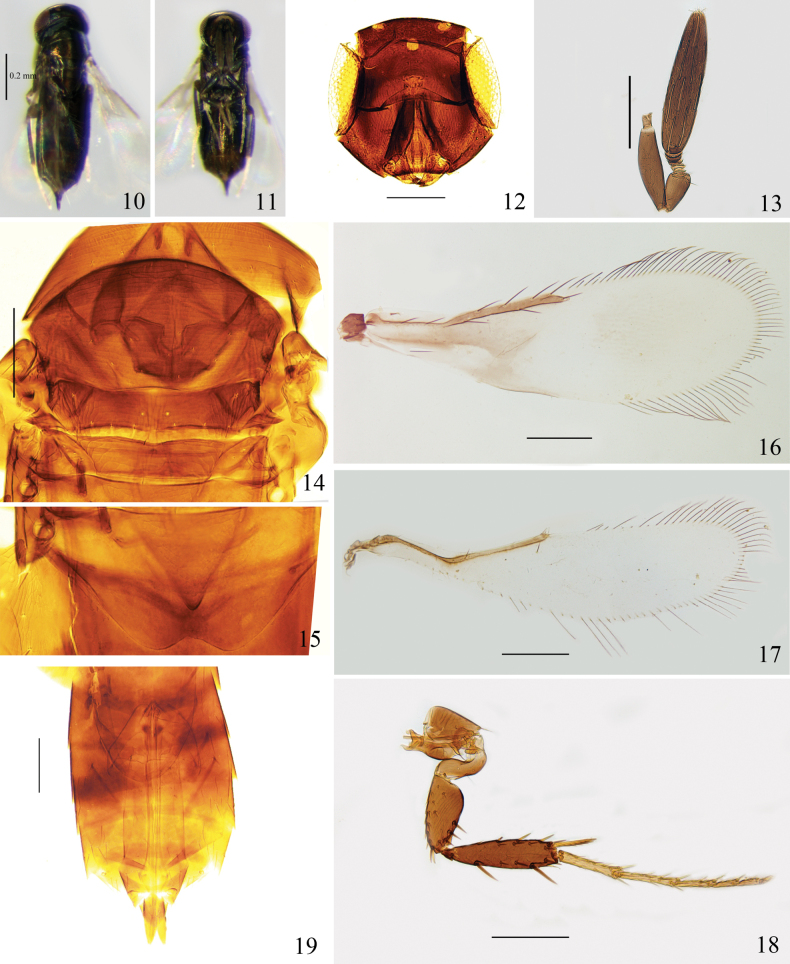
*Chartocerus niger*, ♀. **10.** Body, dorsal view; **11.** Body, ventral view; **12.** Head, frontal view; **13.** Antenna; **14.** Mesosoma; **15.** Propodeum and Mt_1_; **16.** Fore wing; **17.** Hind wing; **18.** Mid leg; **19.** Metasoma. Scale bars: 100 μm (except Fig. 10).

**Female** (Figs 10, 11). Body length 0.6–0.8 mm.

***Colour*.** Generally similar to *C. menglaensis*. Fore wing infuscate basally, with a transverse hyaline area as in Fig. 15; hind wing hyaline.

***Head*** (Fig. 12). In frontal view, height as long as width, mostly smooth, with weakly lineolate sculpture on lower face. Vertex 0.7× the width of head. Eye 0.5× as high as head. Mandible with two teeth. Antenna (Fig. 13) with four anelli; scape 3.8–3.9× as long as wide; pedicel 2.1–2.4× as long as wide; clava 4.7× as long as wide, 1.8–2.3× as long as scape.

***Mesosoma*** (Fig. 14). Pronotum, mesoscutum, and anterior of metanotum transversely imbricate; lateral sides of mesoscutellum and axillae reticulate. Mesoscutum 3.0× as long as mesoscutellum, with 10 setae posteriorly, and a pair of long setae near posterolateral corner. Mesoscutellum with 7 setae along posterior margin, the distance of scutellar sensilla 0.3× the anterior width of mesoscutellum. Metanotum a little shorter than mesoscutellum. Mesopostphragma extending to posterior of Mt_4_.

***Wings*.** Fore wing (Fig. 16) 2.9× as long as wide, with longest setae of marginal fringe 0.3× as long as width of disc, seta M1 absent. Hind wing (Fig. 17) 4.0–4.3× as long as wide, bearing a seta posterior to the apex of marginal vein, posterior margin slightly rounded, with longest setae of marginal fringe 0.4× wing width.

***Leg.*** Mid leg (Fig. 18) with basitarsus 0.5× as long as tibia, and basitarsus obviously longer than (1.2–1.4× as long as) mesotibial spur, mesotibial spur with 6 teeth.

***Metasoma*.** Mt_1_ (Fig. 15) with median portion concaved upward. Ovipositor (Fig. 19) originating from anterior of Mt_3_, 2.5× as long as mesotibia, 1.7× as long as metatibia. Third valvula 1.0× as long as mesobasitarsus.

##### Hosts.

Unknown from this study; see UCD (2025) for detailed records.

##### Distribution.

China (Yunnan) [**new record**], Russia, France, Italy, Spain, North Africa, Egypt, USA, Antilles, Argentina, Bermuda, Brazil, Jamaica, Puerto Rico (UCD 2025; Abd-Rabou and Evans 2016).

##### Comments.

The materials fit well with the description of *C. niger* by Abd-Rabou and Evans (2016). This is the first report of this species from oriental region and the Chinese fauna. Due to the original and subsequent description of the species were quite simple, here we provide more morphological information and some digital photographs for reference.

#### ﻿ Signiphora flavella

Taxon classificationAnimaliaHymenopteraSigniphoridae

Girault, 1913

1CBB79A8-EED6-57B7-827F-E8A04574A6EE

Figs 20, 21

Signiphora flavella Girault, 1913: 214. Lectotype ♀, USNM, not examined.

##### Note.

Complete synonymy and citations, see Woolley and Dal Molin (2017).

##### Material examined.

• 2 ♀, Yunnan Province; Xishuangbanna; Mengla County; Menglun Town; 21°53.62'N, 101°18.25'E; 523 m a.s.l.; 29 April. 2019; Z.-l. Bai, Z.-g. Chen, C. Wang, Y.-f. Tong, H. Yu leg.; LFNU Sc20210707, Sc20210708 [on slides]; • 2 ♀, Yunnan Province; Xishuangbanna; Mengla County; Menglun Town; 21°53.89'N, 101°16.72'E; 568 m a.s.l.; 12 May. 2019; Z.-l. Bai, Z.-g. Chen, C. Wang, H. Yu leg.; LFNU Sc20210709, Sc20210710 [on slides]; • 3 ♀, Yunnan Province; Xishuangbanna; Mengla County; Menglun Town; 21°54.24'N, 101°15.98'E; 541 m a.s.l.; 13 May. 2019; Z.-l. Bai, Z.-g. Chen, C. Wang, H. Yu leg.; LFNU Sc20210711–Sc20210713 [on slides].

Woolley and Dal Molin (2017) provided detailed descriptions for this species. We report this species from China for the first time and provide some photographs for reference.

##### Hosts.

According to Woolley and Dal Molin (2017), armoured scales (Diaspididae) are common hosts of this cosmopolitan species. For details, see Woolley and Dal Molin (2017) and UCD (2025).

##### Distribution.

China (Yunnan) [**new record**]. Other distributional data see Woolley and Dal Molin (2017).

**Figures 20–21.**
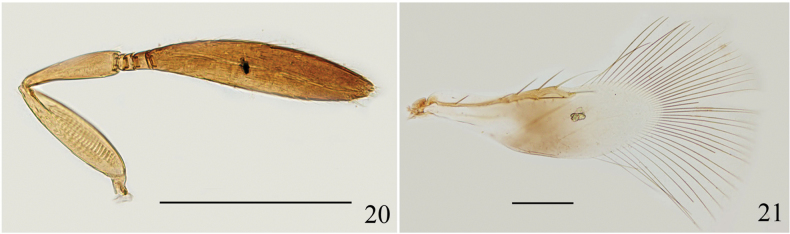
*Signiphora flavella*, ♀. **20.** Antenna; **21.** Fore wing. Scale bars: 100 μm.

## Supplementary Material

XML Treatment for Chartocerus menglaensis

XML Treatment for Chartocerus niger

XML Treatment for Signiphora flavella

## ﻿Additional information

### Use of AI

No use of AI was reported.

### Author ORCIDs

Hui-Feng Zhao https://orcid.org/0000-0003-4243-9671

Hai-Feng Chen https://orcid.org/0000-0001-5838-3700

Ye Chen https://orcid.org/0000-0003-0841-6775

### Data availability

All of the data that support the findings of this study are available in the main text.
